# Temporal lobe necrosis: a dwindling entity in a patient with nasopharyngeal cancer after radiation therapy

**DOI:** 10.1186/1758-3284-3-8

**Published:** 2011-02-10

**Authors:** Meera Dassarath, Zhongyuan Yin, Jing Chen, Hongli Liu, Kunyu Yang, Gang Wu

**Affiliations:** 1 Cancer Center, Union Hospital, Tongji Medical College, Huazhong University of Science and Technology, Wuhan, Hubei, 430022, PR China; 2 Department of Oncology, Queen Victoria Hospital, Candos, Quatre-Bornes, Mauritius

## Abstract

**Introduction:**

Our objective was to report a case of misdiagnosed temporal lobe necrosis (TLN) in a patient with nasopharyngeal cancer (NPC) after radiation therapy.

**Case Presentation:**

We report a case of a 45 years old Chinese woman who developed moderate to severe headache and dizziness 1 year after 2D radiation therapy for NPC. Subsequent MRI scanning revealed a big enhancing mass in the right temporal lobe. The initial diagnosis was metastatic or intracranial extension of NPC, or a primary intracranial malignancy. She was referred to the neurosurgery department where a maximal surgical resection of the lesion was performed. A diagnosis of TLN was made according to the final histology.

**Conclusion:**

TLN still matters in the IMRT era. The diagnostic quagmire of TLN lies in its close resemblance to neoplasm on clinical presentation and imaging. Reviewing the patient's treatment plan to scrutinize the dose to the temporal lobes is an important prerequisite for diagnosis.

## Introduction

Worldwide, nasopharyngeal cancer (NPC) is most common in South China in the provinces of Guangdong, Guangxi, Fujian, Hunan, and Hong Kong. Its incidence in these regions is 30 per 100,000 compared to just 1 per 100,000 in Europe [1]. Radiation therapy remains the main modality of treatment in NPC patients because of anatomic constraints and a high degree of radiosensitivity of this tumor [2-4]. However, temporal lobe necrosis (TLN) is one of the late side effects of radiation therapy that may be encountered in these patients.

The first case of radiation necrosis was described by Fischer and Holfelder in 1930 in a 45 year old patient treated with radiotherapy for basal cell epithelioma of the temporal region with a total dose of 6840 cGy [5]. Lee et al. reported an incidence of up to 3% of TLN in patients after radiation therapy [6]. This entity was once commonly encountered in the era when 2-D technique was widely used for the treatment of NPC. A significant decline of this complication has been observed since the introduction of Intensity-modulated Radiation Therapy (IMRT). Consequently, more and more physicians are oblivious of this complication due to its present rarity. However, knowledge about the possibility of its occurrence, as a delayed side effect of radiation therapy, is vital as it is associated with high morbidity and mortality. It has been seen to account for 65% of radiation related deaths from NPC in Hong Kong [7,8] and the 5 year survival with temporal lobe necrosis, with or without treatment, has been reported to be around 59% there [6].

Owing to its close proximity to the skull base, the medial parts of bilateral temporal lobes are inevitably included in the target volume. Since the inferior portions of the temporal lobes lie within the portals of radiation therapy, TLN is normally seen to occur in bilateral inferomedial parts of the temporal lobes.

Differentiating this condition from metastatic or primary brain tumor remains a clinical and radiological challenge due to their close resemblance. We hereby report the case of a 45 year old Chinese woman with NPC who received radiation therapy at a local hospital using 2D technique and developed severe radiation induced TLN. The diagnosis of TLN, in her case, was missed twice but was finally established by maximal surgical resection and histological examination, hence further laying emphasis on the reduced awareness and diagnostic dilemma of this entity.

## Case presentation

**Figure 1:**
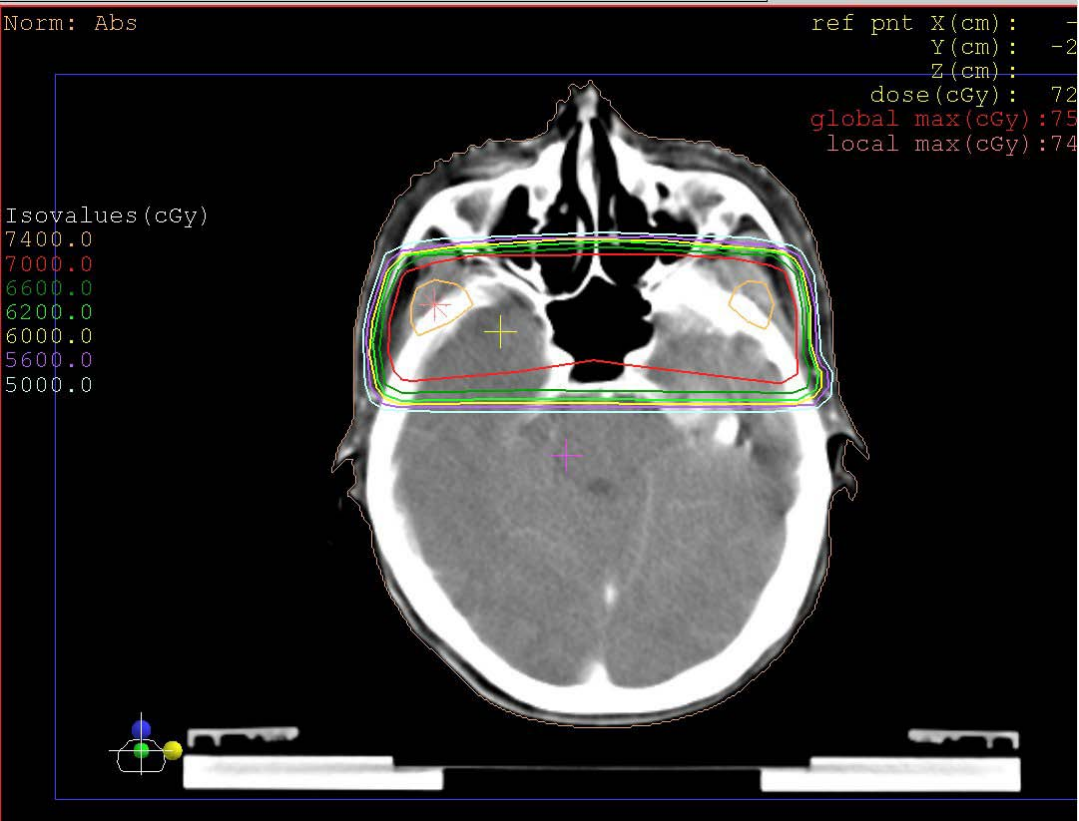
**2D Treatment plan showed that the inferior parts of bilateral temporal lobe received high dose irradiation**.

A 45 year old Chinese lady with a six months history of bilateral nasal obstruction, tinnitus, associated headache and dizziness presented to the local hospital on November 3rd, 2009. Nasoendoscopy revealed a large mass in the nasopharynx, a biopsy of which confirmed non-keratinizing squamous cell carcinoma, WHO type 2.2 [9]. Staging MRI and CT scans showed a large tumor measuring 5 × 3 cm occupying the roof and the posterior wall of the nasopharynx which invaded the inferior part of the clivus. Cross sectional images also revealed bilaterally enlarged lymph nodes in the upper neck. According to the 2002 AJCC staging system [10], she was diagnosed as a NPC patient of stage cT3N2M0. She was treated with 2-phase lateral opposed facial-cervical fields at a 2 Gy daily fraction (Figure 1), 5 times a week to a total dose of 70 Gy with concurrent cisplatin from November 10th to December 31st, 2010. Complete response was achieved after concurrent chemo-radiotherapy. 3 cycles of adjuvant chemotherapy consisting of Cisplatin and 5-fluorouracil was given following concurrent chemo-radiotherapy.

**Figure 2:**
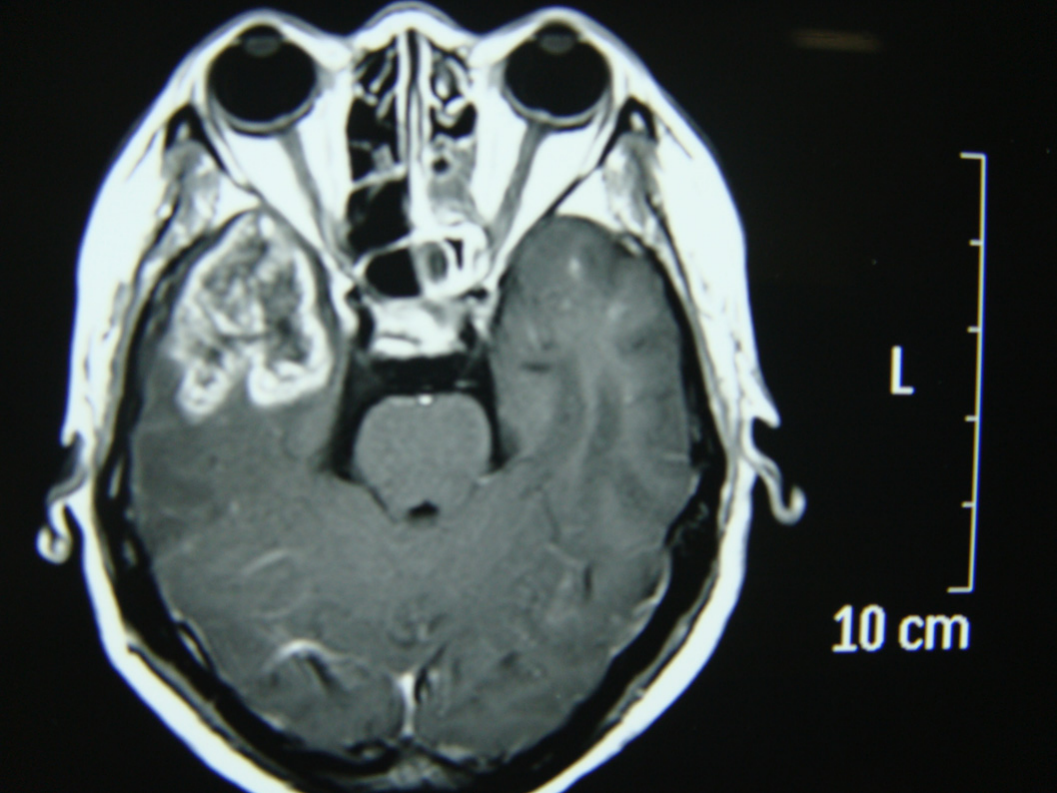
**Axial gadolinium-enhanced T1-weighted images revealed a large heterogeneous mass with circumferential rim enhancement surrounded by extensive edema was found in the inferior part of the right temporal lobe**. Small homogeneous lesions with limited edema could also be seen in the left temporal lobe.

**Figure 3:**
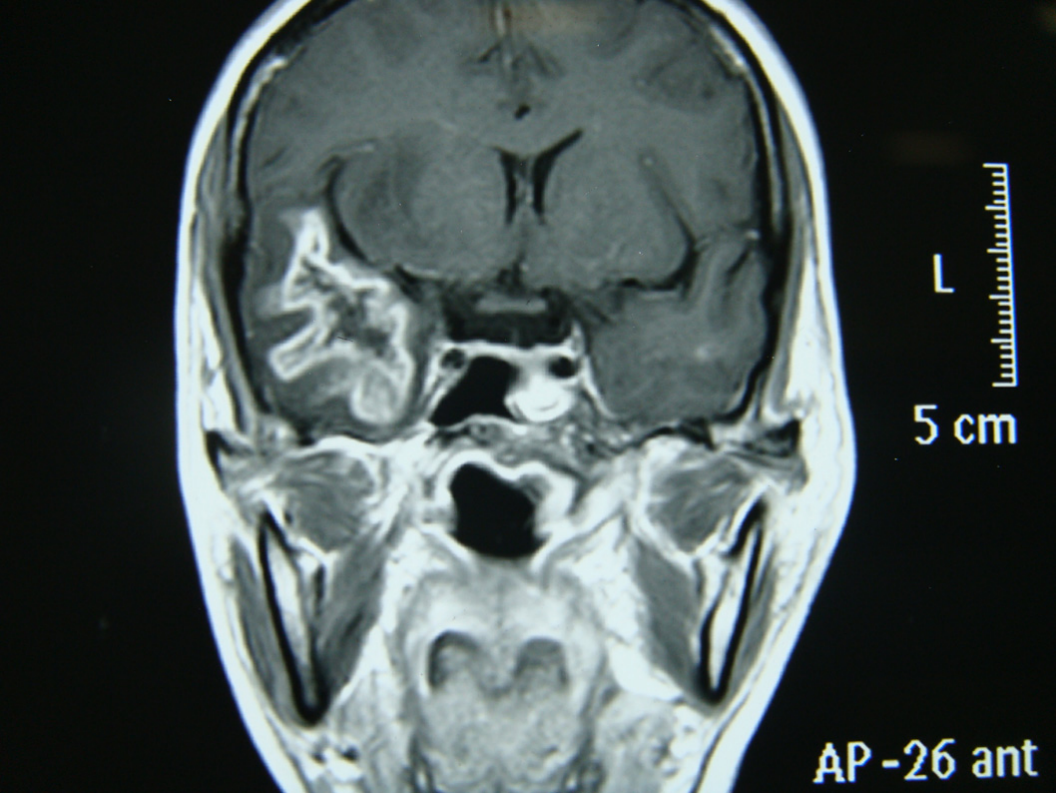
**Coronal images also showed the same findings**.

However, 1 year after radiation therapy the patient developed moderate to severe headache and dizziness. She returned to the local hospital to seek medical intervention for her symptoms on March 2nd, 2010. There was no associated history of fever, blurring of vision, nausea or vomiting. Physical examination was unremarkable. No abnormal neurological signs were noted. Her hematological and biochemical profile as well as her liver function test results were normal. However, Brain MRI scan showed abnormalities in the white matter of bilateral inferior portions of the temporal lobes. A large heterogeneous lesion with circumferential rim enhancement surrounded by extensive edema was found in the inferior part of the right temporal lobe. Small homogeneous lesions with limited edema could also be seen in the left temporal lobe (Figure 2, 3). However, these findings in the contralateral temporal lobe failed to hint the correct diagnosis at the attending physician. Moreover, the MRI also showed associated mild white matter demyelination and cerebral atrophy. Upon assumption that the patient was either a case of primary brain tumor or intracranial extension of NPC, she was referred to the neurosurgery department at local hospital where she was advised to undergo surgery.

**Figure 4:**
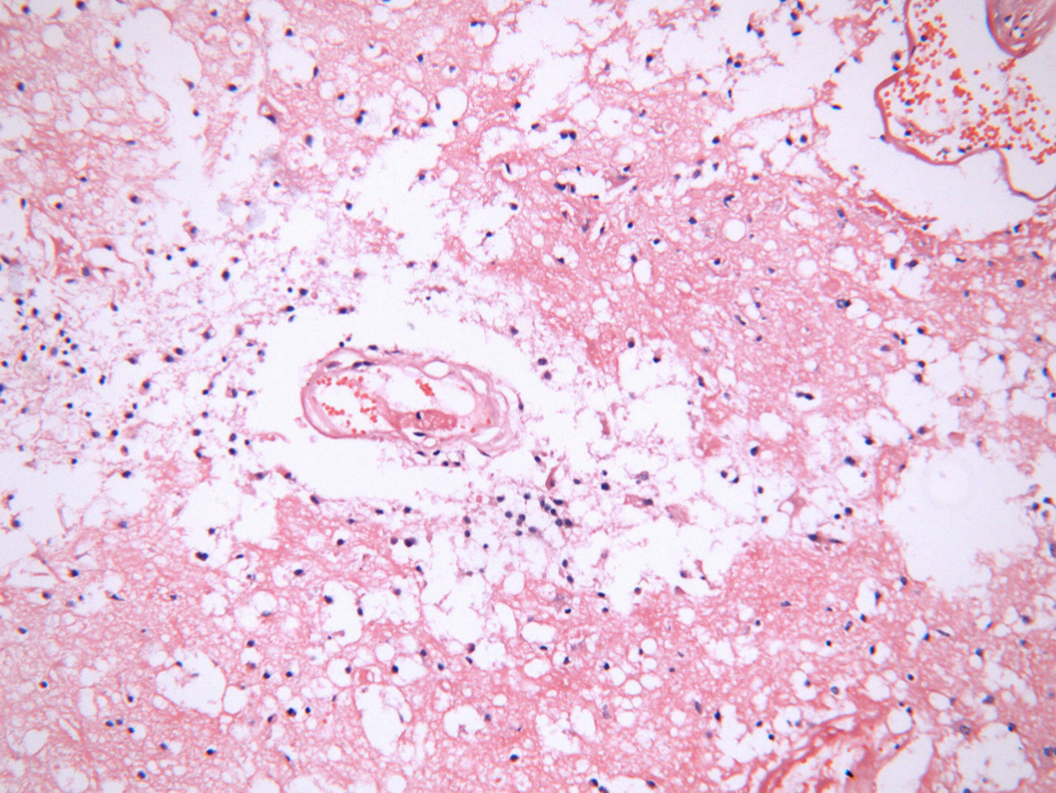
**Histological examination confirmed the absence of neoplastic cells in the resected brain tissues**. Instead, partial liquefactive necrotic tissue with lymphocytic infiltration was noted.

**Figure 5:**
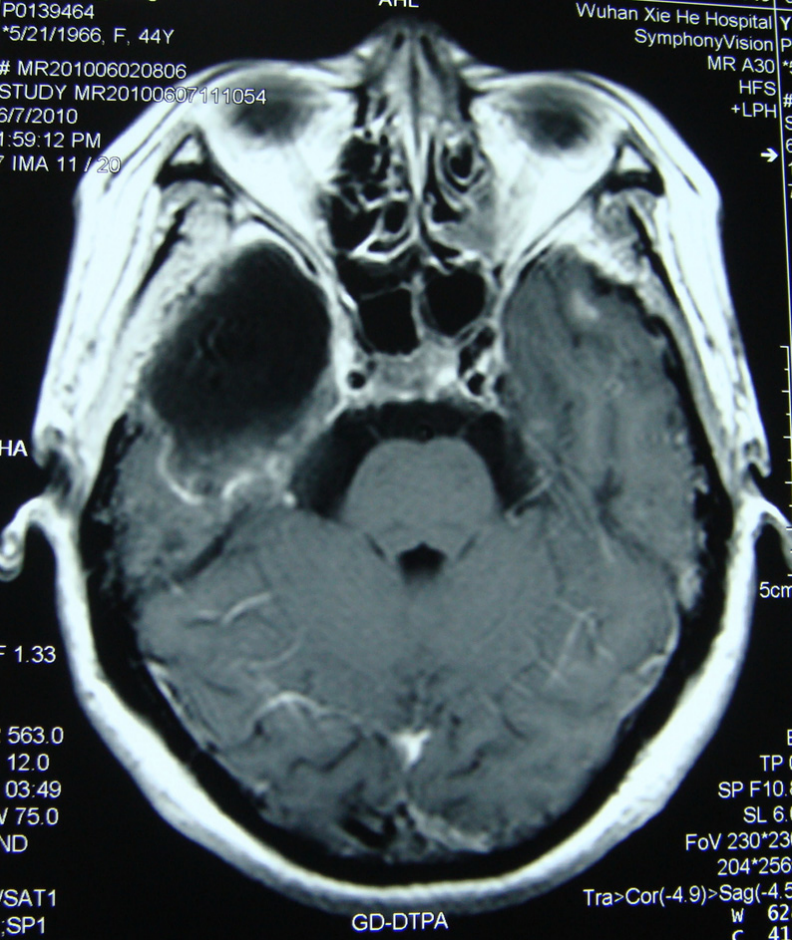
**Postoperative axial gadolinium-enhanced T1-weighted images a big cavity in the lower portion of right temporal lobe with surrounding edema, and several minor enhanced lesions in the counterpart of the left temporal lobe**.

The patient refused surgery or any further treatment. Subsequently, her clinical condition worsened with time. 2 months later she complained of more intense headache especially in the morning with accompanying dizziness, nausea and blurring of vision, which prompted her to return to the neurosurgery department at our hospital in May, 2010. Physical examination showed presence of papilloedema. There was absence of neck rigidity and other focal neurological deficits. Brain MRI re-scanning showed no other new lesions. In keeping with the initial diagnosis of temporal lobe malignancy, the attending neurosurgeon performed a maximal surgical resection of the lesion in the right temporal lobe on May 18th, 2010. However, histological examination confirmed the absence of neoplastic cells in the resected brain tissues. Instead, partial liquefactive necrotic tissue with lymphocytic infiltration was noted. Additionally, there was associated dilatation, congestion and hemorrhage of surrounding blood vessels which corroborated with a diagnosis of radiation induced TLN (Figure 4). Fortunately, her symptoms relieved significantly after resection of the right lesion. Postoperative MRI revealed a big cavity in the lower portion of right temporal lobe with surrounding edema, and several minor enhanced lesions in the counterpart of the left temporal lobe (Figure 5). She presented to our department to seek medical opinion about further treatment for the minor lesions in the left temporal lobe. She was prescribed anticoagulants and high dose vitamins. At present the patient remains well and is in close follow-up 3 months after the TLN resection.

## Discussion

TLN, as a late potential sequalae of radiation treatment in patients with NPC, has experienced a down slope over the past decade. This has occurred with the growing worldwide use of IMRT for treatment of this carcinoma which has provided a potentially therapeutic benefit of dose escalation with reduced toxicity to normal tissues [11]. Symptoms of TLN usually appear one to three years after the last dose of radiation treatment with a silent latent interval noted between the end of treatment and development of radiation-induced necrosis [12]. Four main types of clinical presentations were described in a study by Lee et al. They ranged from complete absence of symptoms to vague features of temporal lobe damage, symptoms of temporal lobe epilepsy or nonspecific characteristics of intracranial lesions. The asymptomatic patients were incidentally diagnosed during follow up with CT or MRI [7]. The clinical progression of our patient was that of non-specific symptoms to overt symptoms of raised intracranial pressure.

Using 2D radiotherapy technique, inferior parts of bilateral temporal lobes almost always receive the same dose as the tumor, especially for patients with locally advanced disease infiltrating the skull base or cavernous sinus. It has been reported that there is a 25% chance of developing TLN within 5 years following use of a total dose of equal to or more than 62.5 Gy [13]. The patient received 70 Gy to her skull base using 2D technique which put her at a high risk of developing radiation necrosis. IMRT, on the other hand, has the advantage of generating complicated 3D dose distributions to conform closely to the target volume and the beam intensity can be optimized using computer algorithms. It has been reported that IMRT provided excellent tumor target coverage and allowed the delivery of a high dose to the target with significant sparing of nearby critical normal tissues [14]. Hence, it plays an important role in decreasing radiation-induced injuries in patients with NPC [15].

The differential diagnoses of TLN include intracranial extension of NPC, second primary intracranial neoplasm, cerebral metastasis, meningeal spread and brain abscess [16]. Differentiation of tumor progression and radiation injury after radiation therapy is indispensable for appropriate treatment [17]. CT, MRI and PET-CT are all useful tools for TLN diagnosis, but none is specific. The characteristic features on MRI include mass effect, vasogenic edema and contrast enhancement. However, MRI alone cannot reliably discriminate tumor from radiation induced necrosis [18], even though the latter can be associated with specific patterns of enhancement such as "soap bubble" or "Swiss cheese" enhancement patterns [19]. The use of functional imaging like the FDG-PET and the proton MR spectroscopy has shown some advantages in differentiating the entities. But presently, they have not been widely used due to their high cost or complicated techniques. A definitive diagnosis is obtained from surgical exploration and biopsy or removal of the necrotic mass [16], but this is not routinely justified. It may not strike the doctor to include TLN as one of the differential diagnoses if he does not review the history and treatment plan of the patient. By carefully correlating the history, the findings on physical examination, the laboratory investigations with the features on imaging scans like brain MRI and CT, a correct working diagnosis can be confidently reached without resorting to biopsy [20-22].

In our case report, a brain abscess can be excluded as our patient had few symptoms and signs to support an infectious disease. Ruling out hematogenous spread of NPC is easy, as it is very rare and would unlikely be present bilaterally [23], while tumor extension is usually associated with erosion of the skull base. Hence, radiation induced necrosis as diagnosis for our patient is supported by the fact that the symptoms developed 6 months after treatment, there was no history of associated fever and the physical findings were consistent with mass effect of the lesion, and last but not the least, the lesions on MRI were bilateral, involving the inferior portions of the temporal lobes, which are usually included in the portals of radiation. However, in our case, this diagnosis may have evaded the attending doctor due to its dwindling existence and close similarity to brain tumor and failure to correlate the bilateralism of the lesions with the treatment plan.

Traditional treatment for TLN includes the use of steroids, hyperbaric oxygen, antiplatelets, anticoagulants, high dose vitamins and surgery, but all have shown a limited efficacy [12,24]. Steroids have been used to provide prompt symptomatic relief and help in retarding the pathologic process [25]. Surgery is used as the last resort palliative measure in patients with significant increase in intracranial pressure or in those who have progressive neurologic deficits despite steroids or other medical therapy. However, in NPC patients who usually have bilateral temporal lobe involvement, surgery can be hazardous. The possible risk of Kluver Bucy syndrome is of concern when considering bilateral temporal lobectomy [7]. The advent of bevacizumab, a humanized monoclonal antibody that inhibits vascular endothelial growth factor (VEGF) has paved the way to possibilities of reversing the pathogenesis of TLN and providing permanent results. Evidence is now available that justifies the consideration of its use in the treatment of radiation necrosis secondary to treatment of head and neck cancers [26-28].

## Conclusion

Although TLN has been dwindling since the introduction of IMRT, it should always be kept in mind when encountering temporal lobe lesions in NPC after radiation therapy. The diagnostic quagmire of TLN lies in its close resemblance to neoplasm on clinical presentation and imaging appearance. This can be achieved by combining the history, physical examination findings, with abnormalities seen on imaging tools like MRI.

## Consent

Written informed consent was obtained from the patient for publication of this case report and accompanying images. A copy of the written consent is available for review by the Editor-in-Chief of this journal.

## Competing interests

The authors declare that they have no competing interests.

## Authors' contributions

KY and GW have made substantial contributions to conception and design, and acquisition, analysis and interpretation of data. MD, ZY, HL, KY have been involved in drafting the manuscript or revising it critically for important intellectual content. All authors read and approved the final manuscript.
